# Plasma optical modulators for intense lasers

**DOI:** 10.1038/ncomms11893

**Published:** 2016-06-10

**Authors:** Lu-Le Yu, Yao Zhao, Lie-Jia Qian, Min Chen, Su-Ming Weng, Zheng-Ming Sheng, D. A. Jaroszynski, W. B. Mori, Jie Zhang

**Affiliations:** 1 Key Laboratory for Laser Plasmas (Ministry of Education), Department of Physics and Astronomy, Shanghai Jiao Tong University, Shanghai 200240, China; 2 Collaborative Innovation Center of IFSA (CICIFSA), Shanghai Jiao Tong University, Shanghai 200240, China; 3 SUPA, Department of Physics, University of Strathclyde, Glasgow G4 0NG, UK; 4 Department of Physics and Astronomy, University of California, Los Angeles, California 90095, USA

## Abstract

Optical modulators can have high modulation speed and broad bandwidth, while being compact. However, these optical modulators usually work for low-intensity light beams. Here we present an ultrafast, plasma-based optical modulator, which can directly modulate high-power lasers with intensity up to 10^16^ W cm^−2^ to produce an extremely broad spectrum with a fractional bandwidth over 100%, extending to the mid-infrared regime in the low-frequency side. This concept relies on two co-propagating laser pulses in a sub-millimetre-scale underdense plasma, where a drive laser pulse first excites an electron plasma wave in its wake while a following carrier laser pulse is modulated by the plasma wave. The laser and plasma parameters suitable for the modulator to work are based on numerical simulations.

## Abstract (web summary)

Optical modulators are easily damaged by high-intensity pulses. Here, the authors propose a method for directly modulating high-power laser light across a broad spectral range using a wave generated in a sub-millimetre-scale underdense plasma by a second laser.

## Graphical abstract

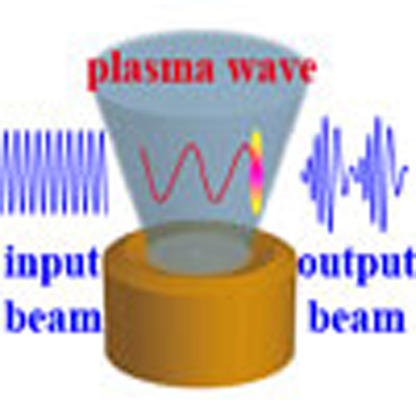

Optical modulators are key components for manipulating optical signals, which are widely used in scientific and industrial applications. For example, high-speed compact electro-optic modulators (EOMs) are essential for data communications^1^,^2^,^3^,^4^. EOMs can alter the fundamental characteristics (that is, amplitude, frequency, phase and polarization) of a light beam in a controllable manner, by making use of electro-optic effects to change the refractive index of a material when an external radio-frequency electric field driver is applied. Thanks to the rapid development of the field of radio-frequency photonics^5^,^6^ together with advanced material and microfabrication technologies^3^,^4^,^7^, the modulation speed of EOMs has dramatically increased from megahertz to 100 gigahertz (refs 8, 9, 10) over the past decade. However, it is still very challenging to successfully achieve terahertz (THz) speed for EOMs using current technologies due to the speed limitation of the high-voltage driver^1^. On the other hand, the rather low optical damage threshold^11^,^12^ and low bulk laser damage threshold of EOMs^13^ severely limit their applications in the high-intensity regime. For example, a state-of-the-art commercially available magnesium-oxide-doped LiNbO_3_ modulator can only handle input light power of 10^2^ mW level and corresponding light intensity at ∼10^2^ W cm^−2^ (ref. 14).

Currently commercial high-power laser systems can deliver peak powers up to petawatts, which can be focused to realize laser intensities from 10^15^ to 10^21^ W cm^−2^. The interaction of such high-intensity laser beams with matter is not only of fundamental interest, but also shows prospects of various applications, such as high-harmonic generation^15^, THz radiation generation^16^,^17^,^18^, plasma-based particle accelerators and light sources^19^, laser fusions^20^, and laboratory astrophysics^21^and so on. With such high-power lasers, it has been reported that plasma-based devices have unique advantages in manipulating intense lasers because they have no damage threshold. Typical plasma-based optical devices include plasma channels for the guided propagation of intense laser pulses over many Rayleigh lengths^22^,^23^, plasma mirrors to improve the temporal contrast of intense laser pulses^24^,^25^, plasma gratings to compress intense laser pulses^26^, plasma lens to focus intense lasers^27^, plasma Raman amplifiers to boost the laser power to the multipetawatt regime or higher^28^,^29^,^30^ and plasma polarization switching for modulating THz electromagnetic waves^31^.

In this article, we show a novel ultrafast all-optical plasma-based modulator that can directly modulate the spectrum of intense laser pulses to an extreme broad bandwidth, with a modulation speed of tens of THz and a damage threshold of 10^16^ W cm^−2^ level. Because of the ultrafast modulation speed and ultrahigh damage threshold, the plasma optical modulator opens a way to efficiently modulate laser pulses in the high-intensity regime. Such highly modulated intense laser pulses may bring a few new physics and applications associated with intense laser–matter interactions. For example, it may be used to produce strong THz radiation via optical rectification as the laser pulses have bandwidth in the THz range^32^. Another possibility is to produce ultrabright X-ray sources via laser interaction with atoms^33^, since the modulated spectrum by our plasma modulator can be well extended to the mid-infrared regime^34^. The deeply modulated laser pulse exhibits ultrabroad bandwidth, which can suppress the growth rate of the stimulated Raman scattering instability, highly important for laser fusions^35^,^36^.

## Results

### Concept of plasma optical modulators

**Figure 1:**
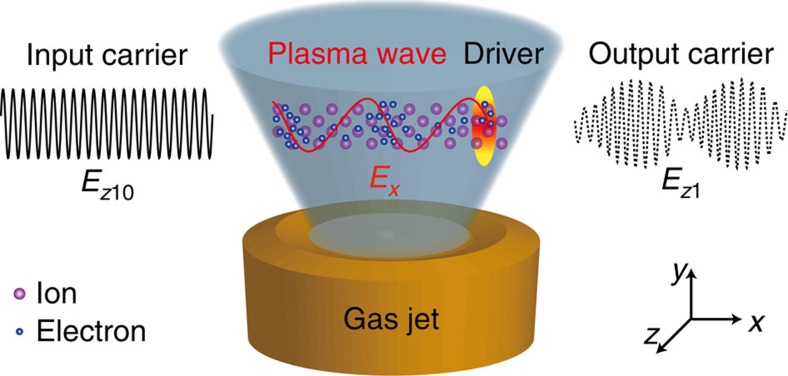
Schematic of a plasma optical modulator. A linearly polarized laser driver propagates (along the *x* direction) in an underdense gas, ionizing the gas and exciting an electron plasma wave in its wake due to the charge separation. A carrier laser co-propagates behind the drive laser, with its amplitude and frequency simultaneously modulated by the plasma wave. Here *E*_*z*10_, *E*_*x*_ and *E*_*z*1_ are the close-up of the electric fields of the unmodulated input carrier, the plasma wave and the modulated output carrier, respectively.

The concept of the plasma optical modulator is illustrated in Fig. 1: a linearly polarized femtosecond intense drive laser pulse propagates in a sub-mm-scale gas, forming an underdense plasma via field ionization. Meanwhile, the ponderomotive force of the laser pulse drives the plasma electrons out of its path. Because the plasma ions are much heavier (by a factor of at least 1,836), they barely move and remain unshielded. The resultant pattern of alternating positive and negative charge-separation fields behind the laser driver is a plasma wave (also called laser wakefields), which has been well-described theoretically^19^ and measured experimentally^37^. The wave oscillates at the plasma frequency *ω*_p_, where 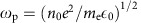, with *n*_0_ the ambient electron plasma density, 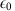 the permittivity of free space, and *m*_e_ and *e* the electron rest mass and charge, respectively. For the purpose of optical modulation, the plasma wave is driven at a moderate amplitude. A picosecond carrier laser pulse (with an arbitrary polarization direction) co-propagates behind the drive laser with a delay of several plasma wavelengths. The amplitude and frequency of the carrier are simultaneously modulated by the plasma wave during its propagation, generating a number of significant frequency sidebands spaced by the plasma frequency in the frequency domain. The modulation speed *f*_p_ is determined by the plasma frequency *ω*_p_, which can be estimated as 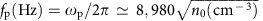, for example, *f*_p_≃28 THz for *n*_0_=10^19^ cm^−3^, which is several orders of magnitude faster than the speed of an EOM. Particle-in-cell (PIC) simulations show that such plasma modulators can sustain a carrier intensity up to 10^16^ W cm^−2^, which is several orders of magnitude higher than what conventional EOMs can handle.

### Parameter dependence of modulation strength

**Figure 2:**
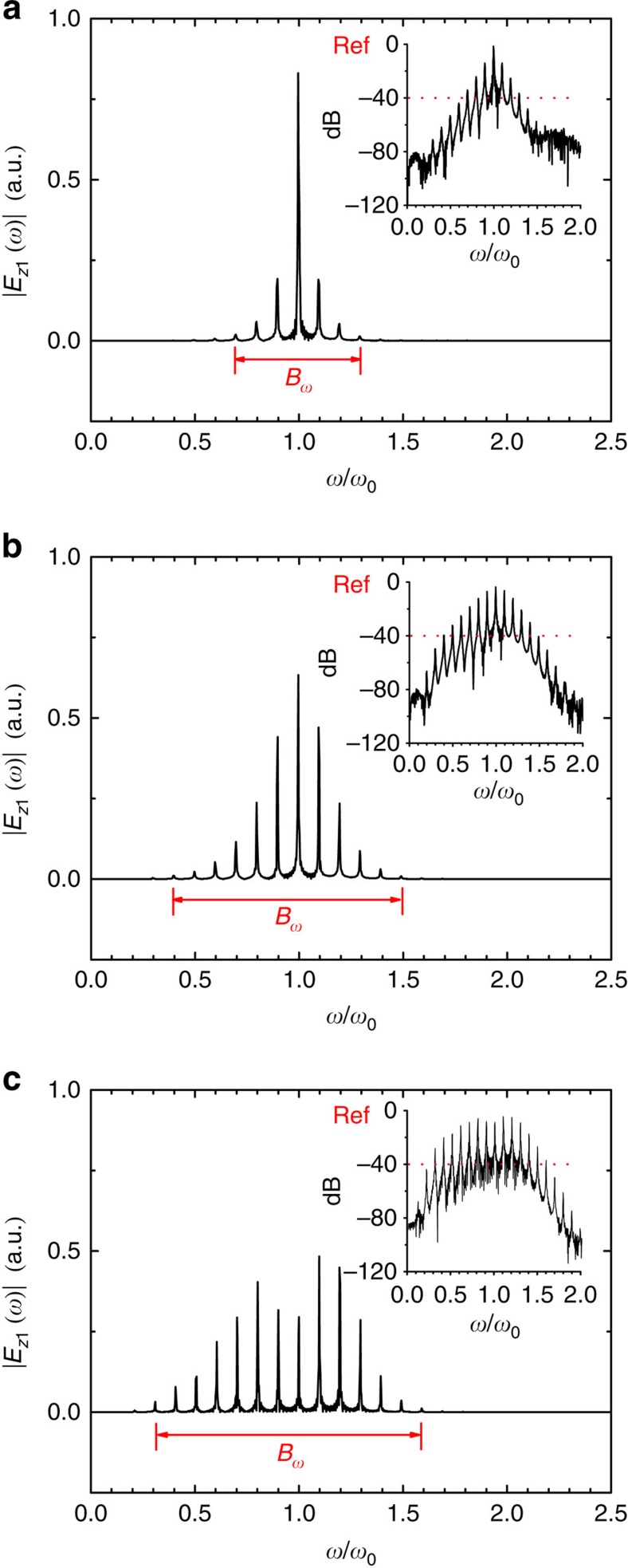
Evolution of the frequency spectrum of the carrier laser. The front of the carrier enters the plasma at *t*=50*T*_L_. (**a**,**b**,**c**) The spectra of the carrier at the propagation time of *t*=250*T*_L_, *t*=400*T*_L_ and *t*=750*T*_L_ (when the carrier completely passes through the plasma), respectively. *T*_L_=2*π*/*ω*_0_ is the laser cycle in vacuum. The insets in each plot show the spectra using logarithmic coordinates, with 0 dB the reference (that is, the unmodulated carrier) amplitude. See the laser–plasma simulation parameters in the Methods.

Figure 2 shows an example case to demonstrate the essential features of the modulation obtained from one-dimensional (1D) PIC simulations. For simplicity, the vacuum wavelengths of the two laser pulses are both 1 μm in the simulations. In practice, an 800-nm Ti:sapphire femtosecond laser pulse can be used to excite the plasma waves, which does not lead to obvious changes of the results presented in the following. The normalized field amplitudes of the driver and the unmodulated carrier are *a*_00_=0.8 and *a*_10_=0.05, respectively, where *a*_*i*0_=*E*_*zi*0_/*E*_*n*_ (*i*=0,1), and *E*_*n*_=*m*_e_*cω*_0_/*e*, with *c* and *ω*_0_ the light speed and angular frequency in vacuum, respectively. For linear polarization, *I*_*i*0_(W cm^−2^)=1.37 × 10^18^*a*_*i*0_^2^/[*λ*_0_(μm)]^2^, with *I*_*i*0_ the peak laser intensity and *λ*_0_=2*πc*/*ω*_0_ the laser wavelength in vacuum. Thus, *a*_00_ and *a*_10_ correspond to the laser intensities of 8.77 × 10^17^ and 3.43 × 10^15^ W cm^−2^, respectively, for 1 μm laser wavelength. Detailed parameters are given in the Methods. The modulated pulse is well described using the analytical model presented in the Methods. It can be expressed as *a*_1_(*t*)=*a*_10_[1+*m* cos(*ω*_p_*t*)] cos[*ω*_0_*t*+*β* sin(*ω*_p_*t*)], where *m* and *β* are the amplitude modulation index and the frequency modulation index, respectively. The mixed amplitude and frequency modulation of a sinusoidal carrier by a simple sinusoidal plasma wave yields a mass of sidebands including both Stokes and anti-Stokes components given by *ω*_*n*_=*ω*_0_±*nω*_p_ (with *n* a nonzero integer and *ω*_p_ as a frequency interval). Note that in the quasi-linear regime of the plasma wave, that is, where the relativistic-electron-mass increase associated with the motion of the plasma electrons can be neglected, the frequency *ω*_p_ can be calculated as *ω*_p_=(*n*_0_/*n*_c_)^1/2^*ω*_0_, with *n*_c_ (cm^−3^)=1.1 × 10^21^/[*λ*_0_(μm)]^2^ the critical plasma density for the corresponding incident laser wavelength *λ*_0_. The spectral bandwidth is defined as *B*_*ω*_=2(*β*+1)*ω*_p_ (ref. 38), where *β* depends on the amplitude of the drive pulse and the plasma density, in addition to the plasma length.

For high fidelity, we only count the significant sidebands with the amplitudes larger than 1% (−40 dB) of the amplitude of the unmodulated carrier^38^. Therefore, the spectral bandwidth of the modulated carrier can be calculated by estimating the number of significant sidebands. As shown in Fig. 2, the higher-order sidebands gradually grow with the laser–plasma interaction time. When the carrier pulse completely passes through the plasma, the maximum significant sidebands for the anti-Stokes and Stokes components are *ω*_+6_ and *ω*_−7_, respectively, giving a bandwidth of *B*_*ω*_=13*ω*_p_=1.3*ω*_0_, accounting for the fact that *ω*_p_=0.1*ω*_0_ for *n*_0_/*n*_c_=0.01. It is also noted that the sideband spectrum of a mixed modulation is asymmetrical due to the superposition of the sideband components of both amplitude and frequency modulations. The simulation results are in good agreement with the prediction of the analytical model given in the Methods. In this example, the amplitude and frequency modulation indices can be estimated^38^ as *m*=(*a*_10,max_−*a*_10,min_)/(*a*_10,max_+*a*_10,min_)=0.42, and *β*=*B*_*ω*_/(2*ω*_p_)−1=5.5, respectively. Note that *β*≫1 corresponds to broadband modulation. The energy transmission rate of the carrier through plasma is ∼94.3% in this example.

**Figure 3:**
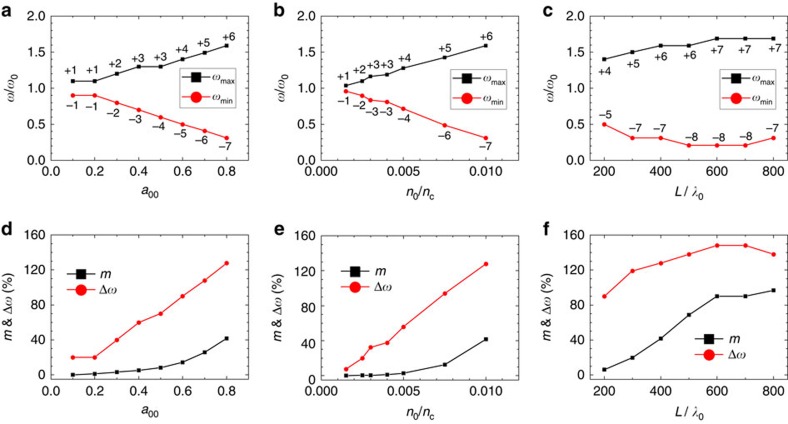
Performance metrics of the plasma optical modulator. (**a**–**c**) The −40 dB sidebands versus (**a**) the driver amplitude, (**b**) the plasma density and (**c**) the plasma length. (**d**–**f**) The −40 dB fractional bandwidth Δ*ω* and the amplitude modulation index *m* versus (**d**) the driver amplitude, (**e**) the plasma density and (**f**) the plasma length. The other laser–plasma parameters are the same as in Fig. 2.

We find the modulation is effective for a wide range of laser–plasma parameters. Figure 3 shows the −40 dB cutoff sidebands, the corresponding fractional bandwidth (Δ*ω*=*B*_*ω*_/*ω*_0_), and the amplitude modulation index *m*, as a function of the driver intensity, the plasma density and the plasma length. When the driver amplitude is relatively small (for example, *a*_00_=0.1), the modulation is quite weak so that the spectrum only consists of the first-order sidebands. By increasing the driver amplitude, the field strength of the plasma wave is enhanced, and subsequently the modulation indices become larger, leading to higher-order sidebands and a wider bandwidth. The similar scaling law exists when increasing the plasma density. Therefore, by properly increasing the drive laser intensity and the plasma density, one can extend the spectrum of the modulated carrier to the mid-infrared regime in the low-frequency side (or the Stokes waves). We note that the growth of the bandwidth is relatively insensitive to the increase of the plasma length after certain distance, which implies a saturation of modulation. As shown in Fig. 3d–f, the amplitude modulation index *m* gradually grows with the increase of the driver intensity or the plasma density. When increasing the plasma length, *m* first grows and then saturates at the 100% level, which indicates that the carrier breaks up into a train of short pulses, and each of these short pulses has a width on the order of the plasma wavelength. According to Fig. 3d–f, Δ*ω* and *β* have similar dependence on the driver intensity, the plasma density and the plasma length as *m*. In the simulation results given above, we have limited the plasma wave excitation to the quasi-linear regime (when the driver laser amplitude *a*_00_≲1). This avoids possible occurrence of curved plasma wave fronts and plasma wave breaking, so that the carrier laser pulse can be modulated efficiently.

**Figure 4:**
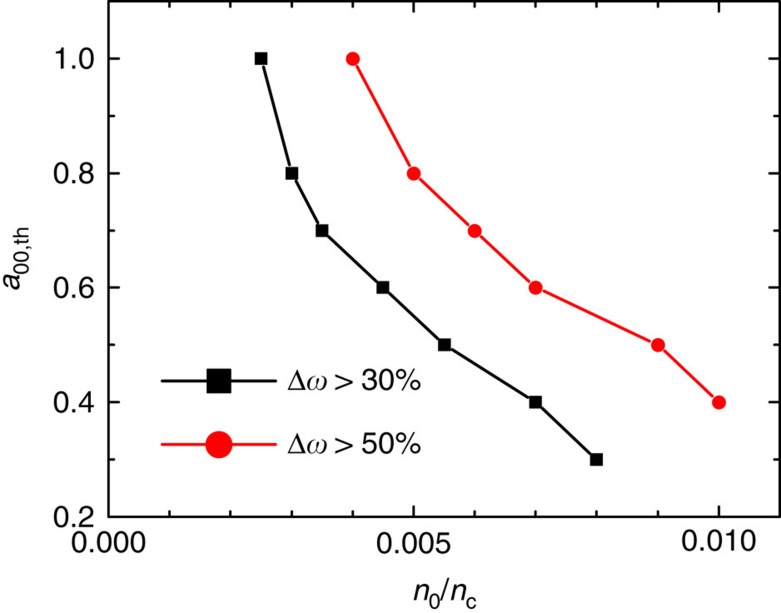
Constraint on the drive laser amplitude and the plasma density for broad bandwidth generation. Threshold driver amplitude versus the plasma density for generating broadband carriers with fractional bandwidths over 30 and 50%. The laser-plasma parameters are the same as in Fig. 2.

For certain applications, it is important to know the parameter range for broad bandwidth generation. Figure 4 illustrates the parameter constraint for generating carrier pulses with ultrabroad bandwidths (for example, Δ*ω* ⩾30%), which presents a series of simulations where the threshold for the driver amplitude *a*_00,th_ is scanned for a given plasma density *n*_0_. In general, a broader bandwidth can be achieved at a higher plasma density even if a lower driver intensity is adopted.

In passing, we mention that, even though the frequencies of the drive laser pulse and the carrier laser pulse can be different in a certain range and the time delay between them can also be arbitrary within a picosecond, the plasma optical modulator requires that the two pulses co-propagate, that is, there is almost no frequency modulation when they counter-propagate.

## Discussion

So far we have discussed the spectrum development of the carrier laser pulse as a function of the drive laser amplitude, the plasma density, and the plasma length. One question still to be answered is the maximum carrier laser intensity allowed in the plasma modulator. A previous study has shown that the resultant pulse train can amplify the field strength of the plasma wake to a wave-breaking level if the initial intensity of the carrier laser is high enough^39^. We also find the remarkably enhanced plasma waves when the intensity of the pulse train is on the same order of the driver intensity (for example, 10^17^ W cm^−2^ level). This can result in severe distortion of the plasma wave and considerable energy loss of the carrier laser to plasma wave excitation as well as electron trapping and acceleration (see Supplementary Fig. 1 and Supplementary Note 1). As a consequence, the frequency modulation of the carrier laser is suppressed. Therefore, the maximum intensity of the carrier laser should be well below 10^17^ W cm^−2^ (for example, at 10^16^ W cm^−2^ level) to realize an excellent performance of the plasma optical modulator.

The maximum allowed pulse duration of the carrier laser for effective modulation may be interesting for some particular applications. This depends on the life time of the electron plasma waves, which is determined by the collisional damping, Landau damping and phase mixing^40^,^41^. Typically the initial electron plasma temperature *T*_e_ is over 10 eV and the effective *T*_e_ is over 100 eV when considering the electron quiver motion in the carrier laser with intensity ∼10^16^ W cm^−2^, which leads to a time scale of over 10 ps for the collisional damping under the plasma electron density of ∼10^19^ cm^−3^. The Landau damping time is much longer than the collisional damping time in the present case when the plasma wave is driven at moderate amplitudes. The phase mixing due to the ion motion is the key responsibility for the plasma wave decay since it occurs on a much shorter time scale of 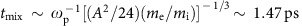 (ref. 40) when a high-Z gas such as argon is used for the plasma wave excitation, where 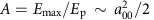 is the normalized amplitude of the plasma wave, with *E*_p_=*cm*_e_*ω*_p_/*e* and *m*_i_ the ion mass. PIC simulations show that the plasma wave starts to decay around 1.65 ps due to the phase mixing, which is in good agreement with the analytical model. The maximum pulse duration for the effective modulation is around 3 ps for the laser–plasma parameters under consideration (see Supplementary Fig. 2, Supplementary Fig. 3 and Supplementary Note 2).

**Figure 5:**
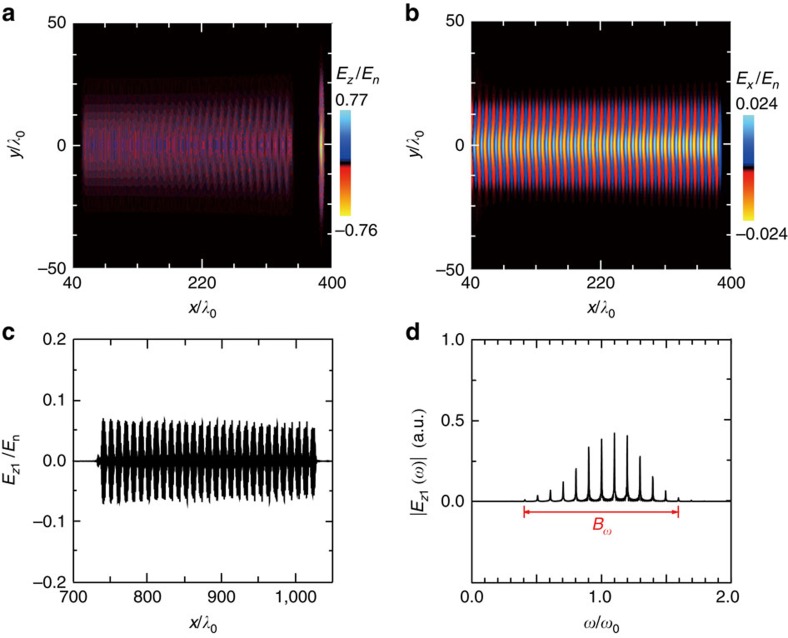
Two-dimensional simulation results. Snapshots of (**a**) the electric fields of the two laser pulses, (**b**) the longitudinal electric field of the plasma wave, at *t*=392*T*_L_, (**c**) the on-axis electric field of the carrier, and (**d**) the corresponding frequency spectrum of the field, when it completely passes through the plasma. Laser-plasma simulation parameters are given in the Methods.

Another issue is the spot sizes of the laser pulses. As we have shown above, the laser pulses need to propagate over a distance of about 1 mm without significant transverse spreading. One needs to take relatively large spot sizes so that the corresponding Rayleigh lengths 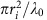 are long enough, with *r*_*i*_ (*i*=0,1) the spot sizes of the two laser pulses. Meanwhile, self-focusing will occur when the driver power *P* exceeds a critical power *P*_c_, with *P*_c_ (GW)=17.4(*ω*_0_/*ω*_p_)^2^. For linear polarization, *P*/*P*_c_=(*ω*_p_*r*_0_*a*_00_)^2^/(32*c*^2^) (ref. 19). To avoid strong self-focusing within 1 mm, the spot size of the driver cannot be too large, either. Two-dimensional (2D) simulations show that the optimal modulation can be achieved for 1≲*P*/*P*_c_≲2. An example of 2D simulation is given in Fig. 5. Detailed parameters are given in the Methods. In this example, the driver power is *P*/*P*_c_=1.18 and weak self-focusing occurs during the propagation. As shown in Fig. 5a, the maximum amplitude of the driver increases by 10% (from *a*_00_=0.7 to 0.77) at a propagation distance of 392*λ*_0_. The excited plasma wave retains the quasi-1D structure, and keeps quite stable during propagation, which is advantageous for the modulation process. It is noted that the driver spot leads to transverse inhomogeneity of the plasma wave, resulting in transverse inhomogeneity of the modulation. By reducing the driver intensity, the corresponding spot size can be increased, and hence, the transverse uniformity of the modulation can be improved.

The technical essentials of realizing the proposed plasma modulators are well within current capabilities. First, the plasma wave excitation by an ultrashort pulse is a well-known technique, which has been widely adopted for laser wakefield accelerators (LWFAs)^19^. For the application to plasma optical modulators, the required amplitude of the plasma wave can be much smaller than that for LWFAs, implying that only moderate drive laser power, such as a few terawatts, is required. Second, the frequencies of the carrier pulse and the drive pulse and the time delay between them are relatively flexible, indicating that the experimental configuration is simpler as compared with some other experiments involving two colliding lasers such as Raman amplification^30^, superradiant amplification^42^ and colliding laser pulses injection in LWFAs^43^. A test experiment of the plasma optical modulator could be carried out with a carrier laser pulse (for example, Nd:YVO_4_, 1 μm, ∼15 mJ, ∼1 ps) delayed with respect to a drive laser pulse (for example, Ti:sapphire, 0.8 μm, ∼150 mJ, ∼30 fs), co-propagating in a 1-mm-long helium gas with a density of ∼10^19^ cm^−3^. The time delay between the two laser pulses can be controlled in a timescale of hundreds of femtoseconds.

In summary, we have illustrated a novel application of the plasma wave as a unique optical modulator for intense lasers. It relies on two co-propagating laser pulses in a short underdense plasma: a driver with a typical intensity ∼10^17^ W cm^−2^, which propagates in the plasma and excites a plasma wake, and a carrier, which propagates behind the driver by several plasma wavelengths. Both the amplitude and frequency of the carrier are modulated by the plasma wave, leading to an ultrabroad bandwidth in its spectrum that extends to the mid-infrared range. The modulation speed is in the THz regime. Compared with the low-damage threshold of the conventional EOMs, the plasma modulator allows the carrier intensity as high as up to 10^16^ W cm^−2^. In addition, the plasma modulator offers excellent performance control by changing the driver intensity, the plasma density and the plasma length. The required experimental conditions for such plasma modulators are within current technical capabilities.

## Methods

### Mathematical model for mixed modulation

Physically, the modulation of the carrier laser pulse by an electron plasma wave is similar to that found for an intense laser propagation in plasma via stimulated Raman forward scattering (coupled with the self-modulation instability)^44^,^45^. The latter leads to a spectrum of Stokes and anti-Stokes waves when the laser pulse has a duration longer than a plasma wavelength. The evolution of the amount of Stokes/anti-Stokes modes can be described by photon acceleration and deceleration^46^,^47^,^48^. The dependence of the spectral modulation on the plasma wave amplitude, the plasma density and the interaction time discussed in the above section also qualitatively agrees with the previous theories. The difference between the spectral modulation described in the previous theories and here is that our plasma optical modulator enables the spectral modulation of the carrier laser to be well controlled and to be developed much more efficiently.

The carrier pulse is modulated in the amplitude and frequency by the electron plasma wave, that is, a mixed modulation. Its temporal structure can be written as^49^


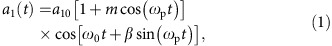


assuming that the excited plasma wave is a simple sinusoidal oscillation, with the normalized axial electric field *E*_*x*_/*E*_p_=(*E*_max_/*E*_p_) cos(*ω*_p_*t*) (ref. 19). Here *a*_10_ is the normalized amplitude of the unmodulated carrier. For a linearly polarized sinusoidal driver with an optimal pulse length for plasma wave excitation (that is, the pulse length approximate to the plasma wavelength), 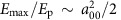, yielding the amplitude and frequency modulation indices 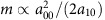 and 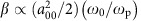, respectively. These two parameters also depend on the interaction time or the plasma length as shown in the simulation results given in Fig. 3. Using simple trigonometrical transformations and a lemma of Bessel function and *J*_−*n*_(*β*)=(−1)^*n*^*J*_*n*_(*β*), *a*_1_(*t*) can be written as


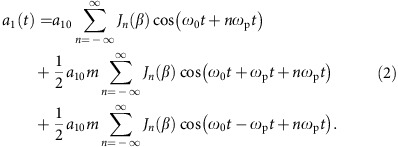


It is obvious that the spectrum of *a*_1_(*t*) primarily consists of three components: the central frequency *ω*_0_ that corresponds to the unmodulated carrier, and the two first-order sidebands *ω*_±1_=*ω*_0_±*ω*_p_ resulting from the modulation process. The amplitudes of the frequency components can be characterized by the expansion in a series of *n*th-order Bessel function *J*_*n*_. By taking Fourier transformation of *a*_1_(*t*), and considering the *k*th-order frequency component with *k* a positive integer, we can get the amplitudes of the upper sideband (*ω*_+*k*_=*ω*_0_+*kω*_p_) and the lower sideband (*ω*_−*k*_=*ω*_0_−*kω*_p_) in the spectrum as follows:


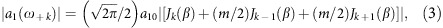



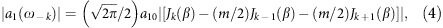


for *ω*_±*k*_>0. From equations (3) and (4), it is straightforward to see that the amplitude of the lower sideband is not equal to the amplitude of the corresponding upper sideband, leading to an asymmetrical sideband spectrum. For a weak frequency modulation (0<*β*<<1), the modulation index is so small that the spectrum essentially consists of *ω*_0_ and only one set of sidebands *ω*_±1_, with the amplitudes of 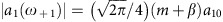, and 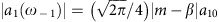. For a large modulation index (*β*>1), there will be a number of significant sidebands spanning over a broad frequency range.

It is worthwhile to mention that when the excited plasma wave is nonlinear, there are high-harmonic components of the plasma oscillations. This leads to an additional frequency modulation at harmonics of the plasma frequency, which are superimposed on the sidebands discussed above, contributing to more energetic sidebands. This nonlinear effect has been included in our self-consistent PIC simulation results presented above.

### PIC simulations

Simulations have been carried out using the code OSIRIS^50^. In the 1D simulations (for example, in Fig. 2), the temporal profile of the drive pulse is 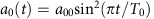, with 0≤*t*≤*T*_0_ and *T*_0_=10*T*_L_. The carrier pulse, which is delayed by 40*λ*_0_ from the driver, has a duration of *T*_1_=303*T*_L_. It has a similar profile as the driver at its leading and trailing edges, and a plateau of 283*T*_L_ in between. The amplitudes of the driver and the carrier are *a*_00_=0.8, *a*_10_=0.05, respectively. The trapezoid-shaped plasma has a length of 400*λ*_0_ with a plateau of 380*λ*_0_, located between *x*=10*λ*_0_ and *x*=410*λ*_0_. The initial plasma electron density in the plateau region is set to be *n*_0_/*n*_c_=0.01. For laser-driven plasma waves, typically the initial (photoionized) electron plasma temperature is set to be 10 eV. The simulation box size is 800*λ*_0_ with 20 macro-particles per cell. The resolution of the computational grid is Δ*x*=*λ*_0_/40. At *t*=0, the front of the driver enters the simulation box. In the 2D simulation (Fig. 5), the amplitude of the driver is *a*_00_=0.7 and the spot sizes of the driver and the carrier are *r*_0_=14*λ*_0_ and *r*_1_=17*λ*_0_, respectively. The trapezoid-shaped plasma has a length of 700*λ*_0_. Other laser–plasma parameters are the same as the 1D simulations. The simulation box size is 1,100*λ*_0_ × 100*λ*_0_ with four macro-particles per cell. The resolution of the computational grid is Δ*x*=*λ*_0_/32 and Δ*y*=*λ*_0_/20.

### Data availability

The data that support the findings of this study are available from the corresponding authors upon request.

## Additional information

**How to cite this article:** Yu, L.-L. *et al*. Plasma optical modulators for intense lasers. *Nat. Commun.* 7:11893 doi: 10.1038/ncomms11893 (2016).

## Supplementary Material

Supplementary Information Supplementary Figures 1-3, Supplementary Note 1-2 and Supplementary Reference
