# Supplementary material for: Plasma optical modulators for intense lasers
Source: Nat Commun. 2016 Jun 10;7:11893. doi: 10.1038/ncomms11893 (PMC4906397; doi:10.1038/ncomms11893)
Supplement: Supplementary Information — Supplementary Figures 1-3, Supplementary Note 1-2 and Supplementary Reference [file ncomms11893-s1.pdf]

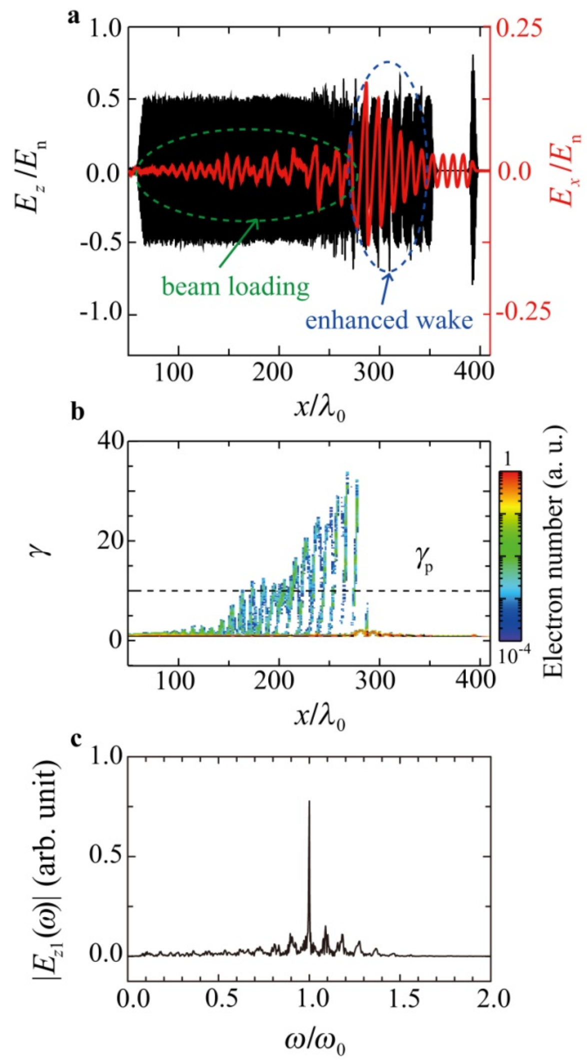

Supplementary Figure 1. The threshold of the input carrier intensity. Snapshots of (**a**) the electric fields of the two lasers (E_z) and the excited plasma wake (E_x), (**b**) the electron energy, and (**c**) the frequency spectrum of the carrier when it completely passing through the plasma. The carrier amplitude here is $a_{10} = 0.5$, and other laser-plasma parameters are the same as in Fig. 2 in the main manuscript.

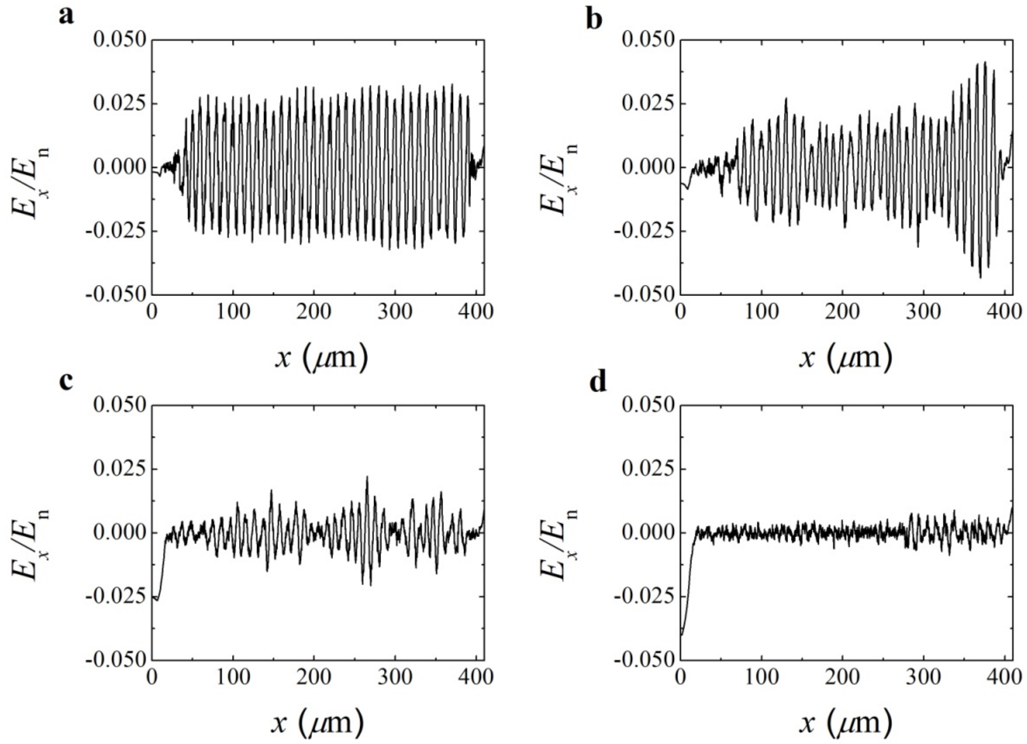

Supplementary Figure 2. The evolution of the plasma wave. Snapshots of the electric fields of the plasma wave at (**a**) t=1.98 ps, (**b**) t=3.3 ps, (**c**) t=4.62 ps, and (**d**) t=5.94 ps. The argon gas is used for the plasma wave generation, and the ion motion is included in the simulation. Other laser-plasma parameters are the same as in Fig. 2 in the main manuscript.

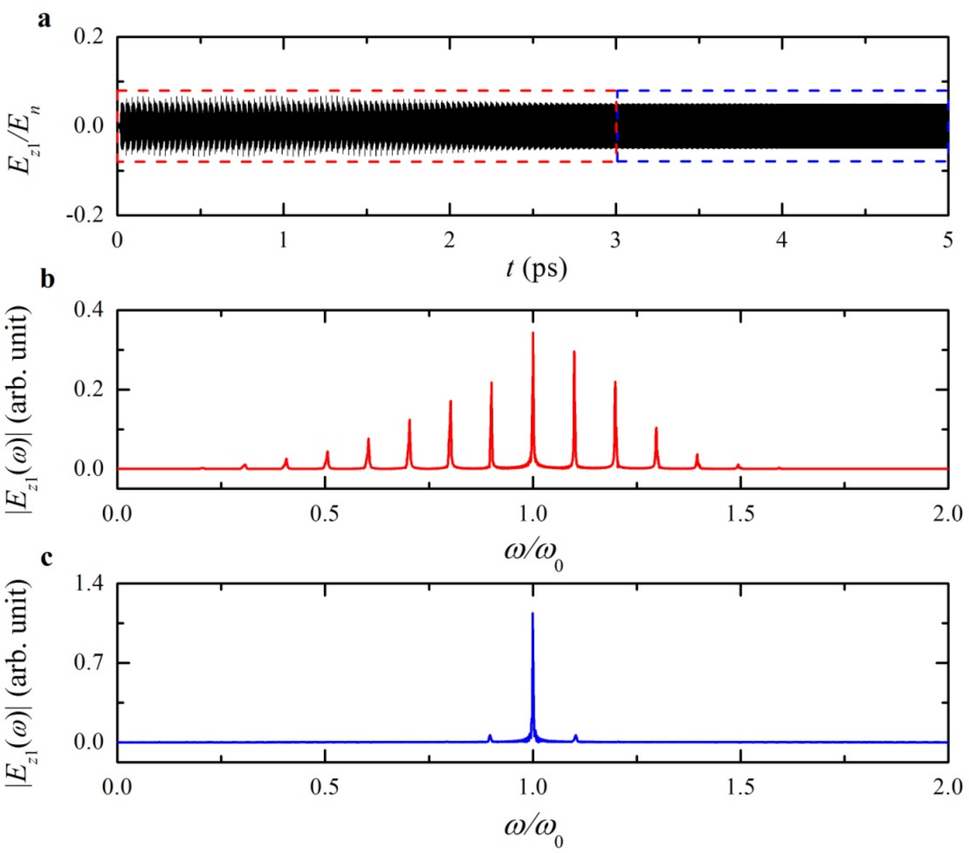

Supplementary Figure 3. The modulation of the 5 ps long carrier laser pulse. a, The electric fields of the carrier laser pulse when it completely passing through the plasma. **b**, The frequency spectrum of the leading part (red rectangle area in **a**) of the carrier laser pulse. **c**, The frequency spectrum of the trailing part (blue rectangle area in **a**) of the carrier laser pulse. Other laser-plasma parameters are the same as in Fig. 2 in the main manuscript.

Supplementary Note 1: Simulating the threshold for the input carrier laser intensity.

When the intensity of the carrier laser pulse is high enough, the electron plasma wave driven by the drive laser will be modified significantly. In particular, at later time when a pulse train is formed, the plasma wave will be amplified to wave breaking when the intensity of the pulse train is on the same order of the drive intensity (at 10^{17} W cm^{-2} level). As shown in Supplementary Fig. 1, the front of the carrier is first modulated by the plasma wave, and then split into several short pulses with the width on the order of the plasma wavelength. These short pulses resonantly excite plasma waves, which enlarge the plasma wave generated by the driver to the wave breaking regime. Significant fraction of the background plasma electrons can be trapped and accelerated as the field of the plasma wave approaches the wave breaking amplitude, as long as they satisfy $\gamma \geq \gamma_{\rm p}$. Here $\gamma = \sqrt{1+u^2}$ is the Lorentz factor of the normalized fluid momentum $\mathbf{u} = \mathbf{p}/m_{\rm e}c$, while $\gamma_{\rm p} = (1-\beta_{\rm p}^2)^{-1/2}$ is the Lorentz factor of the normalized phase velocity of the plasma wave $\beta_{\rm p} = v_{\rm p}/c$. The space-charge field produced by the accelerated electrons significantly modifies the field of the plasma wave, which is often referred to as beam loading[1] and results in severe distortion of the plasma wave. As a consequence, the modulation of the following of the carrier is suppressed, leading to much weaker sidebands compared with the sidebands in Fig. 2c in the main manuscript. Moreover, in this case the frequency interval becomes smaller (i.e., $\omega_{\rm p}/\sqrt{\gamma}$) due to the relativistic motion of the electrons. Simulations show that, for the parameters under consideration ($a_{00} = 0.8$, $n_0/n_{\rm c} = 0.01$), strong suppression of the modulation occurs for $a_{10} \geq 0.3$. Therefore, the maximum intensity of the carrier should be kept below 10^{17} W cm^{-2}. Reducing the driver intensity or the plasma density may help to ameliorate beam loading effects.

Supplementary Note 2：Simulating the maximum allowed pulse duration of the carrier laser for effective modulation.

As discussed in the main manuscript, the maximum pulse duration of the carrier laser for effective modulation depends on the lifetime of the plasma wave exited by the driver. Supplementary Fig. 2 shows the evolution of the plasma wave when using a high-Z gas (e.g., argon) for the plasma wave generation. In this simulation, the drive laser pulse with the amplitude $a_{00} = 0.8$ propagates in the Ar gas, ionizes the Ar to Ar^{8+}, and excites a plasma wave in its wake. It is clearly seen that the plasma wave severely decays after lasting for ~ 3 ps due to the phase mixing caused by the ion motion, resulting in a strong suppression of the modulation of the carrier laser pulse. As shown in Supplementary Fig. 3, when a 5 ps long carrier laser pulse propagates in the plasma, only the leading part (~ 3 ps) is effectively modulated, while the modulation of the trailing part is suppressed due to the plasma wave decay. Therefore, the maximum pulse duration for the effective modulation is around 3 ps for the laser-plasma parameters under consideration.

Supplementary Reference

1. Esarey, E., Schroeder, C. B. & Leemans, W. P. Physics of laser-driven plasma-based electron accelerators. *Rev. Mod. Phys.* **81**, 1229-1285 (2009).
